# 

**Published:** 1885-03

**Authors:** 


### THE BRISTOL

### Medico-Chirurgical Journal.

A JOURNAL OF THE MEDICAL SCIENCES FOR THE WEST OF ENGLAND AND SOUTH WALES.

*PUBLISHED UNDER THE AUSPICES OF*
*THE BRISTOL MEDICO-CHIRURGICAL SOCIETY.*

EDITED BY
J. GREIG SMITH,
M.A., F.R.S.E.

"Scire est nescire, nisi id me

Scire alius sciret."

SURGEON GENL'S OFFICE
LIBRARY.
105605

*VOL III.*

BRISTOL: J. W. ARROWSMITH;
LONDON: J. & A. CHURCHILL, 11 NEW BURLINGTON STREET.
1885.

WI
BR323

J.W. ARROWSMITH,

*Printer and

Publisher,*

QUAY STREET, BRISTOL.

### CONTENTS OF VOL. III.

---

ORIGINAL PAPERS :— *Page*

Discussion on Intestinal Obstruction at the Bristol Meeting of the Bath and Bristol Branch of the British Medical Association ... ... ... ... ... ... ... ... ... ... ... 1

Wounds of the Fingers, and How to Treat Them. By JOHN KENT SPENDER, M.D. London ... ... ... ... ... ... 73

Cremation. By J. G. DAVEY, M.D. ... ... ... ... ... ... 81

The Border-land of Insanity. By W. B. KESTEVEN, M.D. St. And. ... ... ... ... ... ... ... ... ... ... ... 92

A few Remarks upon Aachen (Aix-la-Chapelle). By E. P. PHILPOTS, M.D. ... ... ... ... ... ... ... ... ... 104

Enlargement of the Spleen. By E. LONG FOX, M.D. ... ... 145

A Method of Expressing Ear-power in Degrees of a Fixed Scale. By the Rev. R. A. CHUDLEIGH, M.A. ... ... 159

Pyrexia : a Retrospect, a Review, and a Forecast. Inaugural Address by W. H. SPENCER, M.A., M.D. Cantab., F.L.S. 217

Notes on some Morbid Appearances in the Brain and Spinal Cord of a Lioness. By. W. B. KESTEVEN, M.D. ... 246

The Relations existing between Facial Neuralgia, Frontal Headache and Dental Caries. By H. BOYLE RUNNALLS, M.R.C.S. ... ... ... ... ... ... ... ... ... ... ... 249

CLINICAL RECORDS :—

Retention and Accumulation of Fæces. By J. E. SHAW, M.B. 43

Mangana Water. By E. LONG FOX, M.D.... ... ... ... ... 47

Compound Comminuted Fracture of the Left Femur, with much Laceration of the Soft Parts and the Knee Joint freely opened. By CHARLES F. PICKERING, F.R.C.S. 48

Pendulous Fatty Tumour of Thigh. By SHIRLEY DEAKIN, F.R.C.S. Eng. ... ... ... ... ... ... ... ... ... ... 51

*Page.*

A Case of Hyperpyrexia following Measles, treated successfully by the Wet-pack and Cold Douche. By CHRISTOPHER ELLIOTT, B.A., M.D. ... ... ... ... ... ... ... ... ... 109

On Diphtheria: with Cases. By ALFRED SHEEN, M.D. ... 113

Case of Attempted Suicide, by Precipitation from Clifton Suspension Bridge. By J. FENTON EVANS, M.B. ... ... 124

Rupture of the Aorta. By AUGUSTIN PRICHARD, F.R.C.S. ... 127

Case of Ruptured Aorta. By J. PAUL BUSH ... ... ... ... 129

Five Cases illustrating the Action of Intra-Pulmonary Injections. By R. SHINGLETON SMITH, M.D., B.SC. ... ... ... ... 164

Case of Rupture of Aorta. By HENRY MARSHALL, M.D. ... 178

Two Cases of Intestinal Polypus. By H. BOYLE RUNNALLS 181

Case of Lumbar Spinal Caries. By HARDING H. TOMKINS ... 184

A Case of Sudden Pulmonary Congestion relieved by Aspiration of the Aorta. By J. DACRE ... ... ... ... ... ... ... 189

History of a Case of Lymphadenosis. By JOHN MEREDITH, M.D. ... ... ... ... ... ... ... ... ... ... ... ... 255

A Case of Addison's Disease. By JAMES E. PRICHARD, B.A., M.B. Oxon. ... ... ... ... ... ... ... ... ... ... 269

NOTES ON BOOKS ... ... ... ... ... ... ... ... ... 52, 131, 197, 274

MEDICAL EXTRACTS ... ... ... ... ... ... ... ... 61, 136, 207, 283

OBITUARY ... ... ... ... ... ... ... ... ... ... ... ... ... 287

### Bristol

### Medico-Chirurgical Journal.

---

The *Journal* when first published received the following favourable notices:—

"The Editor must be congratulated upon having produced so readable a number for his first issue. The type and illustrations are also to be commended, and we trust that these efforts will meet with the success they deserve."—*Lancet.*

"It contains a large amount of original matter, is well illustrated."—*Medical Record* (New York).

"The book is nicely got up as regards type and paper, and we are glad to notice that a goodly number of practitioners in the neighbourhood have become subscribers. The *Bristol Medico-Chirurgical Journal* has made an excellent start under favourable auspices."—*Medical Times and Gazette.*

"It also contains numerous clinical records, many of which are of a very interesting character."—*Liverpool Medico-Chirurgical Journal.*

"The clinical cases are especially good, and form a strong feature of the number."—*Birmingham Medical Review.*

"The present number is so full of interesting matter that we look forward with pleasure to the appearance of the second."—*Edinburgh Medical Journal.*

"The original papers are . . . . followed by a goodly array of 'clinical records,' book notices, and abstracts. We congratulate the Editor, Mr. J. Greig Smith, on the promising start the new journal seems to have made."—*New York Medical Journal.*

---

**It is hoped that Subscribers will do all they can to extend the circulation of the Journal, which "is intended to embody and reflect the science and practice of Medicine and Surgery as it exists to-day in the West of England and South Wales."**

---

### Terms for Advertisements.

| | £ | s. | d. |
|---|---|---|---|
| Whole Page ... ... ... ... ... ... ... ... ... | 1 | 1 | 0 |
| Half Page ... ... ... ... ... ... ... ... ... | 0 | 12 | 6 |
| Quarter Page ... ... ... ... ... ... ... ... ... | 0 | 7 | 6 |

Insets (single leaf), 5/-.

Terms for special places and for serial insertions upon application.

Cases for Binding Vols. I. and II. are now ready, and may be obtained, price 7*d.* each, upon application to J. W. Arrowsmith, Quay Street, Bristol; or the *Journal* will be bound, including Case, for 1/3 per volume.

1885.

### LIST OF SUBSCRIBERS

TO THE

### Bristol Medico-Chirurgical Journal.

*Those marked * are Members of the Society.*

| *Name.* | *Residence.* |
|---|---|
| Ackland, W. H., M.D. St. And. | Bideford. |
| Adams, George | 5 Oakfield Road, Clifton. |
| Adams, James, M.D. Aberd. | Ashburton. |
| *Adams, John Barfield | 3 Selwood Villas, Bristol. |
| Alexander, James, M.D., M.Ch. Dub. | Bishop's Place, Paignton. |
| Alford, George Ernest | 5 Royal Crescent, Weston-super-Mare |
| Alford, Henry J., M.D. Lond. | 20 The Crescent, Taunton. |
| *Andrews, Onslow, M.D. St. And. | 159 White Ladies Road, Bristol. |
| Anstie, Thomas B. | 21 Northgate Street, Devizes. |
| *Atchley, George F., M.B. Lond. | Tyndall's Park, Bristol. |
| Baretti, T. G. L'Enardi | 49 Royal York Crescent, Clifton. |
| *Baron, B. J., M.B., C.M. Ed. | 12 Richmond Hill, Clifton. |
| Batten, Rayner W., M.D. Lond. | 1 Brunswick Square, Gloucester |
| *Beddoe, John, M.D. Ed., B.A. Lond., F.R.S. | 2 Lansdown Place, Clifton. |
| Bennett, W. S. | 12 The Terrace, Penzance. |
| Berry, F. C., M.D., B.Ch. Dub. | Lynton. |
| Bisdée, Alfred J. | Eversleigh, Banwell. |
| *Blacker, A. E. | 7 Unity Street, Bristol. |
| Blacker, E. | Midsomer Norton. |
| *Blagg, Arthur F. | 6 West Mall, Clifton. |
| Blaxall, F. H., M.D. St. And. | Clan Lodge, Bath. |
| Board, Edmund C. | 7 Caledonia Place, Clifton. |
| Bond, Francis T., M.D. Lond. | 1 Beaufort Buildings, Spa, Gloucester. |
| Bousfield, E. C. | 363 Old Kent Road, London. |
| Bower, Ernest D. | 1 Norfolk Terrace, Gloucester. |
| Boyle, Thomas | Newquay. |
| *Boys, Arthur Henry | Lodway Villa, Pill. |
| Broom, John, M.D. Brussels | St. Paul's Road, Clifton. |
| Broster, Alfred, M.D. Giessen | Zetland Road, Bristol. |
| Brown, G. Arthur | Tredegar. |
| *Burder, George F., M.D. Aberd. | 63 Oakfield Road, Clifton. |
| Burges, R. E., M.D., M.Ch., B.A. Qu. Univ. I. | Kettering. |
| Burroughs, E. F. H. | Rook Lane House, Frome. |
| *Bush, J. Paul | 2 Rodney Cottages, Clifton. |
| Cann, Francis M. | Sefton House, Dawlish. |
| Carey, J., M.D. Lond. | 2 Hamdon Villas, Taunton. |
| *Carr, Alexander | Wells Road, Bristol. |
| *Carter, W. F. | Coronation Road, Bristol. |
| *Challacombe, J. P., M.D. Ed. | 143 Cheltenham Road, Bristol. |
| Clark, Thomas | Blair Lodge, Minehead. |
| *Clark, T. E., M.D., C.M. Aberd. | 3 Victoria Square, Clifton. |
| Clarke, Fred. H. | Chillington. |
| Clarke, W. Michell | 2 York Buildings, Clifton. |

### LIST OF SUBSCRIBERS.

| | |
|---|---|
| Clay, R. Hogarth, M.D. Ed. | 4 Windsor Villas, Plymouth. |
| Coathupe, Edwin W. | 7 Clifton Park, Clifton. |
| *Coe, R. W. | 14 Berkeley Square, Bristol. |
| Coker, T. V. | 2 Chesterfield Place, Clifton. |
| Cole, Thomas, M.D. Lond. | 18 Circus, Bath. |
| *Collins, H. W. | Wrington. |
| Colman, Thomas J., M.D. Ed. | Redland Road, Bristol. |
| Colmer, Ptolemy S. H., M.D. Dur. | South Street House, Yeovil. |
| Colthurst, J. B. | Wincanton. |
| Cooke, A. S. | Badbrook House, Stroud. |
| Cooper, Herbert | Rosslyn Hill, Hampstead, London, N.W. |
| *Corbould, George G. | 22 Berkeley Square, Bristol. |
| Cornwall, James | Fairford. |
| Couch, James | Christina Street, Swansea. |
| Cowan, F. S. | 27 Queen Square, Bath. |
| *Crisp, Nathaniel | Chandos House, Keynsham. |
| *Cross, F. Richardson, M.B. Lond. | Chandos Villa, Clifton. |
| *Crossman, Edward | Hambrook. |
| Cunningham, A. G., M.B., C.M. Aberd. | Stapleton Road, Bristol. |
| Currie, Andrew S., M.D. Ed. | Oakfield Villa, Lydney. |
| *Dacre, John | Royal Infirmary, Bristol. |
| Daly, George H., M.D. St. And. | Burley House, Chippenham. |
| *Davey, James G., M.D. St. And. | 4 Redland Park, Bristol. |
| *Davies, D. S., M.B. Lond. | 60 Oakfield Road, Clifton. |
| Davis, Theodore, M.D. Lond. | Beachcroft, Clevedon. |
| Day, Percy Howard | Stalmine, Poulton-le-Fylde. |
| Dening, Edwin | Stow-on-the-Wold. |
| *Dew, Henry R. | 25 Berkeley Square, Bristol. |
| *Dobson, Nelson C. | 27 Victoria Square, Clifton. |
| *Dowson, C. H. | Tyndall's Park, Bristol. |
| *Dowson, W., M.B., M.A. Cantab. | 18 Kingsdown Parade, Bristol. |
| Doyne, R. W. | Southbourne, Oxford. |
| Dunbar, Eliza L. Walker, M.D. Zürich | 9 Oakfield Road, Clifton. |
| Eadon, W. F. Bailey | Hambrook. |
| *Eager, Reginald, M.D. Lond. | Northwoods. |
| Edgeworth, T. F. | 1 Stokes Croft, Bristol. |
| *Elliott, Christopher, M.D. Dub. | 155 White Ladies Road, Bristol. |
| *Elwin, J. Fountain | 17 Clyde Road, Bristol. |
| *Evans, J. Fenton, M.B., C.M. Ed. | Royal Infirmary, Bristol. |
| *Ewens, John | 20 Cotham New Road, Bristol. |
| Fairbanks, W., M.D., C.M. Ed. | Wells. |
| Fasulo, Violetta | Nurses' Institute, Bournemouth. |
| *Fendick, R. George | 3 Claremont Place, Clifton. |
| Fernie, James | Stratton St. Margaret. |
| Ferris and Co. | Union Street, Bristol. |
| Fiddian, Alexander P., M.B. Lond. | 6 Brighton Terrace, Cardiff. |
| Fletcher, Henry Studd | Lydbrook. |
| Forty, D. H. | Wotton-under-Edge. |
| Fox, Arthur E. W., M.B., C.M. Ed. | 16 Gay Street, Bath. |
| *Fox, Bonville B., M.B., M.A. Oxon. | Brislington. |
| *Fox, E. Long, M.D. Oxon. | Church House, Clifton. |
| Fraser, D. A. | Arranmore House, Burnham, Bridgwater. |
| Fraser, Græme B. | 7 Lower Church Road, Weston-s.-Mare. |
| Fry, J. Farrant | 203 St. Helen's Road, Swansea. |
| Fry, John B. | Swindon. |

### LIST OF SUBSCRIBERS.

| | |
|---|---|
| FULLER, J. | Long Ashton. |
| *FYFFE, W. JOHNSTONE, M.D. Dub. | 2 Rodney Place, Clifton. |
| GARDINER, GEORGE | 1 Redcliff Hill, Bristol. |
| *GIBBS, ALFRED N. GODBY | 27 Chandos Road, Bristol. |
| GILES, G. F., M.D. | Lower Redland Road, Bristol. |
| *GLOAG, G. A. | 6 College Green, Bristol. |
| GOODDEN, C. W. | 31 Stapleton Road, Bristol. |
| GOODING, J. CALLENDER, M.D. Ed. | Berkeley Street, Cheltenham. |
| GOODRIDGE, HENRY F. A., M.D. Lond. | 10 Brook Street, Bath. |
| GOSS, T. BIDDULPH | 36 Paragon, Bath. |
| GOWING, B. C. | Avon House, Salisbury. |
| GRACE, ALFRED | Chipping Sodbury. |
| GRACE, EDWARD MILLS | Park House, Thornbury. |
| GRACE, HENRY | Kingswood, Bristol. |
| GRACE, W. G. | 57 Stapleton Road, Bristol. |
| GREEN, F. K. | 3 Gay Street, Bath. |
| GREGORY, GEORGE S. | Vernon Chambers, Southam'n Row, Lond. |
| *GRIFFITHS, L. M. | 9 Gordon Road, Clifton. |
| GROTE, G. W. | 32 Portland Square, Bristol. |
| HARPER, JOSEPH | Bear Street, Barnstaple. |
| *HARRISON, A. J., M.B. Lond. | Guthrie Road, Clifton. |
| *HARSANT, W. H. | 16 Pembroke Road, Clifton. |
| HAWKINS, CÆSAR F. | 7 Berkeley Square, Bristol. |
| HAYMAN, A. G. | 1 Pembroke Road, Clifton. |
| *HEAVEN, JOHN C. | Avonmouth. |
| *HERAPATH, C. K. C. | 11 Brunswick Square, Bristol. |
| *HETLING, H. E. | Stapleton Road, Bristol. |
| HICHENS, JAMES S. | 17 Green Lane, Redruth. |
| HINTON, JOSEPH | Warminster. |
| HOPKINS, H. CULLIFORD | 4 Gay Street, Bath. |
| HOWSE, WILLIAM | 8 London Street, New Swindon. |
| HOWSIN, E. ARTHUR, M.D. St. And. | Stroud. |
| HYATT, BROWNLOW N. | Park View, Shepton Mallet. |
| *IMLAY, G. A., M.B., C.M. Aberd. | 4 Apsley Villas, Bristol. |
| INFIRMARY, THE ROYAL | Bristol. |
| INSTITUTION, LIVERPOOL MEDICAL (*Librarian*, WILLIAM JONES). | |
| *ISAAC, G. WASHINGTON M.B., C.M. Ed. | 8 King's Parade, Clifton. |
| JAMES, J. BOWEN | Woodlands, Aberaman, Aberdare. |
| JENKINS, JOHN HENRY | Liskeard. |
| JESSOP, CHARLES MOORE | 15 Blomfield Terrace, London, W. |
| JONES, EVAN | Ty-mawr, Aberdare. |
| JOYNES, FRANCIS JAMES | Eagle House, Dursley. |
| *KEALL, W. P. | Coronation Road, Bristol. |
| KEMPE, ARTHUR W. | 20 St. Sidwell's, Exeter. |
| KESTEVEN, W. B., M.D. St. And. | Little Park, Enfield, Middlesex. |
| KING, LOUIS J. | 10 Russel Street, Bath. |
| KINNEIR, R. | The Tower House, Malmesbury. |
| KIRKMAN, J. MILLER | Wootton Bassett. |
| LAING, W. A. GORDON, M.B., C.M. Glasg. | Mevagissey. |
| *LANSDOWN, F. POOLE | 19 White Ladies Road, Clifton. |
| *LAWRENCE, A. E. AUST, M.D., C.M. Aberd. | 15 Richmond Hill, Clifton. |
| LAWRENCE, A. G., M.D. St. And. | Chepstow. |
| *LAWRENCE, HENRY | 9 Park Row, Bristol. |
| *LAXTON, HENRY | 12 Apsley Road, Clifton. |
| *LEWIS, J. R., M.B., C.M. Glasg. | 20 Gloucester Road, Bristol. |

### LIST OF SUBSCRIBERS.

| | |
|---|---|
| Liddon, William, M.B. Lond. | Church Square, Taunton. |
| Lloyd, Walter E. | 60 York Road, Bristol. |
| Lodge, John | Keynsham. |
| Logan, Frederic T. B. | Dean Lane, Bristol. |
| Lovell, W. Day | The Abbey, Bradford-on-Avon. |
| Lovell, William Frederick | West End House, Compton Martin. |
| Lowe, T. Pagan | Royal United Hospital, Bath. |
| *Luffman, W. C. | 8 Cotham New Road, Bristol. |
| *Lyons, A. de Courcy, M.B., C.M. Aberd. | Henley Lodge, Yatton. |
| Maclean, J. Campbell, M.B., C.M. Ed. | Swindon House, Swindon. |
| Manning, H. J. | Laverstock House, Salisbury. |
| Marley, Henry F. | The Nook, Padstow. |
| Marsh, Charles J. | Yeovil. |
| Marsh, O. E. Bulwer | 35 Bridge Street, Newport, Mon. |
| *Marshall, Henry, M.D. Ed., F.R.S.E. | 28 Caledonia Place, Clifton. |
| Martin, Theodore | Temple Cloud. |
| Mason, Frederick | 20 Belmont, Bath. |
| Mason, S. B. | 12 Park Terrace, Pontypool. |
| Mason, William | St. Austell. |
| Matthews, Charles E. | Stapleton Road, Bristol. |
| Mayor, T. O. | 40 Apsley Road, Clifton. |
| *Mead, H. Rimmington | Fishponds. |
| Meredith, John, M.D. Ed. | Wellington, Somerset. |
| Milne, John, M.B., C.M. Aberd. | 97 Clarence Road, Bristol. |
| Milward, James | 54 Charles Street, Cardiff. |
| Morgan, John | Langport. |
| Morgan, William, M.D. Aberd. | 16 Edwards Terrace, Cardiff. |
| Morris, J. A. | Caerleon. |
| Moynan, W. Arthur, M.D., M.Ch. Qu. Univ. I. | Wonford House, Exeter. |
| Murray, William, M.B., C.M. Ed. | Staple Hill. |
| Napier, T. W. A., M.B., C.M. Aberd. | Strathwye, Tintern. |
| *Newman, Charles | 19 Portland Square, Bristol. |
| Newstead, James | 9 York Place, Clifton. |
| Newton, Charles John | Oriel Lodge, Cheltenham. |
| Nicholson, T. D., M.D., C.M. Ed. | 2 White Ladies Road, Clifton. |
| Norman, J. H. | Goodleigh, Winkleigh. |
| Norman, J. W. | Edde Cross House, Ross. |
| *Norton, J. A., M.D., C.M. Aberd. | 8 Redcliff Hill, Bristol. |
| *Norton, T. Chalmers | 12 King Square, Bristol. |
| Nourse, W. J. Chichele | Morchard Bishop. |
| Ollerhead, T. J. | Hillbury, Minehead. |
| *Ormerod, Henry | Westbury-upon-Trym. |
| Padley, George | 2 Northampton Terrace, Swansea. |
| Paine, William Henry, M.D. Aberd. | Stroud. |
| Parette, James | 7 College Green, Bristol. |
| Parry, J. H. | 99 Ashley Road, Bristol. |
| *Parson, T. Cooke | 21 White Ladies Road, Bristol. |
| Parsons, Francis J. C. | King Square, Bridgwater. |
| Parsons, J. D. F., M.D. St. And. | 13 Dowry Square, Clifton. |
| Parsons, J. Fred. | Willow Vale, Frome. |
| Pauli, G. C. P. | 240 York Road, Bristol. |
| Pearse, Fred. E., M.D. St. And. | Hall House, Frome. |
| *Penny, W. J. | 42 Caledonia Place, Clifton. |
| Penruddocke, Charles | Winchcombe. |
| Pentland, Young J. | 11 Teviot Place, Edinburgh. |

### LIST OF SUBSCRIBERS.

| | |
|---|---|
| Perkins, E. B. Steele | 21 Magdalen Street, Exeter. |
| Perkins, J. H., M.D. New York | 14 Victoria Square, Clifton. |
| Phelps, W. Harford G., M.D. Aberd. | Weston-super-Mare. |
| Phelps, William H. | 123 Ashley Road, Bristol. |
| Philipps, S. Rees, M.D., M.Ch. Qu. Univ. I. | Wonford House, Exeter. |
| Phillips, E. England | Broadwater House, Southend. |
| Philpots, Edward P., M.D., C.M. Aberd. | Bourne Hall, Bournemouth. |
| *Pickering, C. F. | 12 Berkeley Square, Bristol. |
| Pizey, G. F. P. | Rydal Villa, Clevedon. |
| Potter, Samuel R., M.B., B.A. Dub. | Collumpton. |
| *Pountney, W. E., M.D., C.M. Ed. | 107 White Ladies Road, Bristol. |
| Powell, William, M.B. Lond. | Hill Garden, Torquay. |
| *Prichard, Arthur W. | 31 St. Paul's Road, Clifton. |
| *Prichard, Augustin, M.D. Berlin | 4 Chesterfield Place, Clifton. |
| *Prichard, J. E., M.B., B.A. Oxon. | 243 Cheltenham Road, Bristol. |
| Prideaux, T. Engledue P. | Wellington, Somerset. |
| *Prowse, Arthur B., M.D. Lond. | 4 Rodney Cottages, Clifton. |
| Randolph, Charles | Milverton, Somerset. |
| Rawlings, John Adams | 4 Northampton Terrace, Swansea. |
| Redwood, T. Hall, M.D., C.M., M.A. Dur. | The Lawn, Rhymney. |
| Rogers, George, M.D. St. And. | 6 Portland Square, Bristol. |
| Rossiter, George F., M.B. Lond. | Cairo Lodge, Weston-super-Mare. |
| *Roué, W. Barrett, M.D., M.S. Dur. | 2 Buckingham Place, Clifton. |
| Roxburgh, Robert, M.B. Ed. | 1 Victoria Buildings, Weston-super-Mare. |
| *Rudge, C. King | 145 White Ladies Road, Bristol. |
| *Rudge, H. T. | 5 Colston's Parade, Bristol. |
| Russell, M. W. H. | Royal United Hospital, Bath. |
| Salmon, W. G. | Thornbury. |
| Sanctuary, Thomas, M.D. Ed. | 29 Castle Street, Salisbury. |
| Scott, James, M.B., C.M. Ed. | H.M. Prison, Hyde Road, Manchester. |
| Scott, Richard J. H. | 13 Bladud Buildings, Bath. |
| Searle, George C. | Brixham. |
| Seward, W. J., M.B. Lond. | County Asylum, Colney Hatch, Lond., N. |
| Sewart, J. H. | Lostwithiel. |
| Shapter, Lewis, M.D. Cantab. | The Barnfield, Exeter. |
| *Shaw, J. E., M.B., C.M. Ed. | 11 Lansdown Place, Clifton. |
| Sheen, A., M.D. St. And. | 23 Newport Road, Cardiff. |
| Sheldon, T. Steele, M.B. Lond. | Parkside House, Macclesfield. |
| *Shorland, Walter E. | 189 Cheltenham Road, Bristol. |
| Sincock, J. Bain | Friarn Street, Bridgwater. |
| Skeate, Edwin | 16 Paragon, Bath. |
| *Skelton, Henry | Downend. |
| *Skerritt, E. Markham, M.D., B.S., B.A. Lond. | Richmond Hill, Clifton. |
| *Skinner, Stephen, M.B., C.M. Aberd. | Ferndale, Clevedon. |
| Smith, George | Axbridge. |
| *Smith, George | The Coombe, Westbury-upon-Trym. |
| *Smith, G. Munro | 70 Pembroke Road, Clifton. |
| *Smith, J. Greig, M.B., C.M., M.A. Ab., F.R.S.E. | 16 Victoria Square, Clifton. |
| Smith, J. Sidney, M.D. St. And. | Tiverton. |
| *Smith, R. Shingleton, M.D., B.Sc. Lond. | 9 Richmond Hill, Clifton. |
| Smith, Samuel | 7 Kingsdown Parade, Bristol. |
| Society, Cardiff Medical (*Hon. Sec.*, T. Garrett Horder). | |
| Society, Plymouth Medical (*Hon. Sec.*, C. Whipple). | |
| Society, Royal Medical and Chirurgical | 53 Berners Street, London, W. |
| Somer, James | Broadclyst. |

### LIST OF SUBSCRIBERS.

| | |
|---|---|
| *SPENCER, W. H., M.D., M.A. Cantab. ... ... | 5 Lansdown Place, Clifton. |
| SPENDER, JOHN K., M.D. Lond. ... ... ... | 17 Circus, Bath. |
| STANSFELD, G. M. ... ... ... ... ... ... ... | 19 Victoria Square, Clifton. |
| STEEL, S. H., M.B. Lond. ... ... ... ... ... | Abergavenny. |
| *STEELE, CHARLES, M.D. Dur. ... ... ... ... | 17 Richmond Hill, Clifton. |
| *STEPHENS, LOCKHART E. W. ... ... ... ... | General Hospital, Bristol. |
| *STEWART, JAMES, B.A. Qu. Univ. I. ... ... | Dunmurry, Stoke Bishop. |
| STOCK, G. ... ... ... ... ... ... ... ... ... | Barton Hill Road, Bristol. |
| STOCKWELL, FRED., M.D. Lond. ... ... ... | Bruton. |
| STORRY, FREDERICK W. ... ... ... ... ... | Lansdown, Stroud. |
| *SWAYNE, J. G., M.D. Lond. ... ... ... ... | 74 Pembroke Road, Clifton. |
| *SWAYNE, S. H.... ... ... ... ... ... ... ... | 129 Pembroke Road, Clifton. |
| SYDNEY-TURNER, A. M. ... ... ... ... ... | College Green, Gloucester. |
| TAYLER, GEO. CHRISTOPHER, M.D. Lond. ... | Trowbridge. |
| TAYLOR, FRANK ... ... ... ... ... ... ... | 5 Colston's Parade, Bristol. |
| *TAYLOR, J. ... ... ... ... ... ... ... ... ... | 5 Ashley Road, Bristol. |
| TAYLOR, THOMAS HENRY ... ... ... ... ... | Prospect House, Thornbury. |
| TERRY, HENRY G. ... ... ... ... ... ... ... | 2 Green Park, Bath. |
| THOMAS, A. GARROD, M.D., C.M. Ed. ... ... | 28 Bridge Street, Newport, Mon. |
| THOMAS, R. W. ... ... ... ... ... ... ... | Tregare House, Keynsham. |
| *THOMPSON, GEORGE, M.D. Dur. ... ... ... | City and County Asylum, Stapleton. |
| THOMSON, G. S., M.D. Ed. ... ... ... ... | 8 College Road, Clifton. |
| THOMSON, SPENCER, M.D. St. And. ... ... | Ashton, Torquay. |
| *THURSTON, HUGH C. ... ... ... ... ... ... | Royal Infirmary, Bristol. |
| *TIVY, W. J. ... ... ... ... ... ... ... ... | 8 Lansdown Place, Clifton. |
| TODD, W. J. ... ... ... ... ... ... ... ... | North Petherton. |
| TOSSWILL, LOUIS H., M.B. Cantab. ... ... | 49 Magdalen Street, Exeter. |
| TWINING, A. H. ... ... ... ... ... ... ... | The Knoll, Salcombe. |
| TYLEY, R. P., M.D. St. And. ... ... ... ... | Wedmore. |
| VALENTINE, EDMUND W. ... ... ... ... ... | Somerton, Somerset. |
| WADE, A. LAW, M.D. Dub. ... ... ... ... | Somerset County Asylum, Wells. |
| *WALDO, HENRY, M.D., C.M. Aberd. ... ... | 19 Pembroke Road, Clifton. |
| WALLACE, REV. C. HILL, M.A. Oxon. ... ... | 3 Harley Place, Clifton. |
| WALSH, DAVID H. ... ... ... ... ... ... ... | Ivywell House, Stoke Bishop. |
| WALTER, WILLIAM HENRY ... ... ... ... ... | Knapp Villa, South Petherton. |
| WARD, J. L. W. ... ... ... ... ... ... ... | Victoria Street, Merthyr Tydvil. |
| *WATHEN, J. HANCOCKE ... ... ... ... ... | 18 Richmond Hill, Clifton. |
| WATSON, J. ADAM ... ... ... ... ... ... ... | Chudleigh. |
| WATTERS, GEORGE T. B., M.D., C.M. Ed. ... | Stonehouse. |
| WAUGH, ALEXANDER ... ... ... ... ... ... | Midsomer Norton. |
| *WEATHERLY, LIONEL A., M.D., C.M. Aberd. | Portishead. |
| WEBB, W. H. ... ... ... ... ... ... ... ... | Kingsbridge. |
| *WEBSTER, THOMAS ... ... ... ... ... ... | Redland Hill, Bristol. |
| *WELSH, F. F. ... ... ... ... ... ... ... ... | Rockleaze Point, Stoke Bishop. |
| WHITFELD, W. CLARKE ... ... ... ... ... | 5 St. Ethelbert Street, Hereford. |
| WICKHAM, W. ... ... ... ... ... ... ... ... | Hill House, Tetbury. |
| WICKSTEED, FRANCIS W. S. ... ... ... ... | Chester House, Weston-super-Mare |
| WILLCOX, R. LEWIS ... ... ... ... ... ... | Warminster. |
| WILLETT, M., M.D. St. And. ... ... ... ... | 87 Ashley Road, Bristol. |
| WILLIAMS, WILLIAM HENRY, M.D. Lond. ... | Sherborne. |
| WILTON, JOHN P. ... ... ... ... ... ... ... | 10 College Green, Gloucester. |
| WINTERBOTHAM, L. ... ... ... ... ... ... | Bays Hill, Cheltenham. |
| WISE, N. V. ... ... ... ... ... ... ... ... | Fore Street, Trowbridge. |
| WOODD, H. T. ... ... ... ... ... ... ... ... | Calstock. |
| WORKMAN, C. J., M.D. Ed. ... ... ... ... | Teignmouth. |

### The following Periodicals are received in Exchange with the Bristol Medico-Chirurgical Journal.

### AFRICA.

CAPE COLONY.

*Monthly.*

*The South African Medical Journal.* East London: T. W. Goodwin.

EGYPT.

*Twice a Month.*

*Unione Medica Egiziana.* Alexandria: E. Pergola.

### AMERICA.

ARGENTINE REPUBLIC.

*Monthly.*

*Anales del Circulo Médico Argentino.* Buenos Ayres: J. N. Klingelfuss.

CANADA.

*Monthly.*

*Canada Medical and Surgical Journal.* Montreal: Gazette Printing Company.

PERU.

*Monthly.*

*La Crónica Médica.* Lima: Carlos Prince.

UNITED STATES.

*Weekly.*

*The Medical and Surgical Reporter.* Philadelphia: 115 South Seventh Street.

*The Medical Record.* New York: William Wood & Co.

*The New York Medical Journal.* New York: D. Appleton & Co.

*The Cincinnati Lancet and Clinic.* Cincinnati: 281 West 7th Street.

*The Journal of the American Medical Association.* Chicago: 65 Randolph Street.

*Fortnightly.*

*Philadelphia Medical Times.* Philadelphia: J. B. Lippincott & Co.

*Monthly.*

*The American Practitioner.* Louisville: John P. Morton and Company.

*The Polyclinic.* Philadelphia: P. Blakiston, Son & Co.

*The Saint Louis Medical and Surgical Journal.* Saint Louis: 2622 Washington Avenue.

*The Therapeutic Gazette.* Detroit: George S. Davis.

*The Analectic.* New York: G. P. Putnam's Sons.

*Journal of Cutaneous and Venereal Diseases.* New York: William Wood & Company.

*The American Journal of Ophthalmology.* St. Louis: J. H. Chambers & Co.

*The International Review of Medical and Surgical Technics.* Palatka: 103-105 Reid Street.

*The Obstetric Gazette.* Cincinnati: 281 West 7th Street.

*Quarterly.*

*The American Journal of the Medical Sciences.* Philadelphia: Henry C. Lea's Son & Co.

*Occasionally.*

*Transactions of the New York Academy of Medicine.* New York: 12 West 31st Street.

### ASIA.

INDIA.

*Monthly.*

*The Indian Medical Journal.* Allahabad: The Pioneer Press.

### AUSTRALASIA.

AUSTRALIA.

*Monthly.*

*The Australasian Medical Gazette.* Sydney: L. Bruck.

*The Australian Medical Journal.* Melbourne: Stillwell & Co.

### EUROPE.

BELGIUM.

*Monthly.*

*Bulletin de l'Académie royale de médecine de Belgique.* Brussels: A. Manceaux.

BRITISH ISLES.

*Weekly.*

*British Medical Journal.* London: 161 A Strand.

*Medical Times and Gazette.* London: J. & A. Churchill.

*The Lancet.* London: 423 Strand.

*Monthly.*

*The Birmingham Medical Review.* Birmingham: Cornish Bros.

*The London Medical Record.* London: Smith, Elder, & Co.

*The Medical Chronicle.* Manchester: John Heywood.

*Edinburgh Medical Journal.* Edinburgh: Oliver and Boyd.

*The Glasgow Medical Journal.* Glasgow: Alex. Macdougall.

*The Dublin Journal of Medical Science.* Dublin: Fannin & Co.

*Quarterly.*

*The Asclepiad.* London: Eade and Caulfield.

*Half-yearly.*

*The Liverpool Medico-Chirurgical Journal.* Liverpool: Medical Institution.

*The Retrospect of Medicine.* London: Simpkin, Marshall, & Co.

*Yearly.*

*The Medical Annual.* London: Henry Kimpton.

*The Year-Book of Treatment.* London: Cassell & Company, Limited.

DENMARK.

*Weekly.*

*Hospitals-Tidende.* Copenhagen: Jacob Lund.

FRANCE.

*Three times a week.*

*Gazette des hôpitaux.* Paris: 4 Rue de l'Odéon.

*L'Union médicale.* Paris: 11 Rue de la Grange-Batelière.

*Weekly.*

*Bulletin de l'Académie de médecine.* Paris: G. Masson.

*Gazette médicale de Paris.* Paris: 8 Place de l'Odéon.

*Le Progrès médical.* Paris: 14 Rue des Carmes.

*Monthly.*

*Revue mensuelle de Laryngologie, d'Otologie et de Rhinologie.* Paris: 8 Place de l'Odéon.

GERMANY.

*Twice a Week.*

*Deutsche Medizinal-Zeitung.* Berlin: Eugen Grosser.

GREECE.

*Weekly.*

Γαληνὸς. Athens: N. G. Passare.

HOLLAND.

*Weekly.*

*Nederlandsch Tijdschrift voor Geneeskunde.* Amsterdam: F. van Rossen.

ITALY.

*Monthly.*

*Lo Sperimentale.* Florence: 35 Via degli Alfani.

NORWAY.

*Monthly.*

*Norsk Magazin for Lægevidenskaben.* Christiania: Th. Steens.

PORTUGAL.

*Weekly.*

*A Medicina Contemporanea.* Lisbon: José Antonio Rodrigues & Ca.

RUSSIA.

*Weekly.*

*Vrach.* St. Petersburg: C. Ricker.

SWITZERLAND.

*Monthly.*

*Illustrirte Monatsschrift der ärztlichen Polytechnik und Centralblatt der orthopädischen Chirurgie.* Berne: K. Schmid.

### BOOKS AND PAMPHLETS RECEIVED.

*Lectures on Mental Disease.* By W. H. O. Sankey. Second Edition, with Illustrations. London: H. K. Lewis. 1884.

*Notes on Materia Medica and Pharmacy.* By Frederick T. Roberts. London: H. K. Lewis. 1884.

*Handbook of Diseases of the Eye.* By H. R. Swanzy. London: H. K. Lewis. 1884

*Elements of Surgical Diagnosis.* By A. Pearce Gould. London: Cassell and Co. 1884.

*Intestinal Obstruction.* By Frederick Treves. London: Cassell and Co. 1884.

*Practical Pathology.* By G. Sims Woodhead. Second Edition. Edinburgh: Young J. Pentland. 1885.

*Revival of Ovariotomy.* By Sir Spencer Wells. Reprint. 1884.

*Clinical Observations of the Blood of the Insane.* By S. Rutherford Macphail. Reprint. 1885.

*The Lunacy Law: its Defects; and a Scheme of Reform.* By William R. Huggard. Reprint. 1885.

### MANUALS

FOR

### Students of Medicine.

THIS Series has been projected to meet the demand of Medical Students and Practitioners for compact and authoritative Manuals embodying the most recent discoveries, and presenting them to the reader in a cheaper and more portable form than has till now been customary in Medical Works.

The Manuals contain all the information required for the Medical Examinations of the various Colleges, Halls, and Universities in the United Kingdom and the Colonies.

The Authors will be found to be either Examiners or the leading Teachers in well-known Medical Schools. This ensures the practical utility of the Series, while the introduction of the results of the latest scientific researches, British and Foreign, will recommend them also to Practitioners who desire to keep pace with the swift strides that are being made in Medicine and Surgery.

In the rapid advance in modern Medical knowledge, new subjects have come to the front which have not as yet been systematically handled, nor the facts connected with them properly collected. The treatment of such subjects forms an important feature of this Series.

New and valuable Illustrations are freely introduced. The Manuals are printed in clear type, upon good paper. They are of a size convenient for the pocket, and bound in red cloth limp, with red edges. They contain from 300 to 540 pages, and are published at prices varying from 4s. 6d. to 7s. 6d.

(*For List of Manuals see over.*)

### Manuals for Students of Medicine.

I.—**Elements of Histology.** By E. KLEIN, M.D., F.R.S., Joint-Lecturer on General Anatomy and Physiology in the Medical School of St. Bartholomew's Hospital, London. *Third Edition.* 6s.

II.—**Surgical Pathology.** By A. J. PEPPER, M.B., M.S., F.R.C.S., Surgeon and Teacher of Practical Surgery at St. Mary's Hospital. 7s. 6d.

III.—**Surgical Applied Anatomy.** By FREDERICK TREVES, F.R.C.S., Surgeon to and Lecturer on Anatomy at the London Hospital. 7s. 6d.

IV.—**Clinical Chemistry.** By CHARLES H. RALFE, M.D., F.R.C.P., Assistant Physician at the London Hospital. 5s.

V.—**The Dissector's Manual.** By W. BRUCE CLARKE, M.B., F.R.C.S., and C. B. LOCKWOOD, F.R.C.S., Demonstrators of Anatomy, St. Bartholomew's Hospital Medical School. 6s.

VI.—**Human Physiology.** By HENRY POWER, M.B., F.R.C.S., Examiner in Physiology, Royal College of Surgeons of England. 6s.

VII.—**Materia Medica and Therapeutics: An Introduction to Rational Treatment.** By J. MITCHELL BRUCE, M.D., F.R.C.P., Lecturer on Materia Medica at Charing Cross Medical School, and Physician to the Hospital. 7s. 6d.

VIII.—**Physiological Physics.** By J. MCGREGOR-ROBERTSON, M.A., M.B., Muirhead Demonstrator of Physiology, University of Glasgow. 7s. 6d.

IX.—**Surgical Diagnosis: A Manual for the Wards.** By A. PEARCE GOULD, M.S., M.B., F.R.C.S., Assistant Surgeon to Middlesex Hospital. 7s. 6d.

X.—**Operative Surgery.** By EDWARD BELLAMY, F.R.C.S., Surgeon and Lecturer on Anatomy at Charing Cross Hospital; Examiner in Anatomy, Royal College of Surgeons.

XI.—**Comparative Physiology and Anatomy.** By F. JEFFREY BELL, M.A., Professor of Comparative Anatomy at King's College.

XII.—**Forensic Medicine.** By A. J. PEPPER, M.S., M.B., F.R.C.S., Examiner in Forensic Medicine to the University of London.

XIII.—**Medical Applied Anatomy.** By JOHN CURNOW, M.D., F.R.C.P., Professor of Anatomy at King's College, Physician at King's College Hospital.

*Other Volumes will follow in due course.*

*Cassell & Company, Limited, London; and all Booksellers.*

### Clinical Manuals

FOR

*Practitioners and Students of Medicine.*

**Complete Monographs on Special Subjects.**

THE object of this Series is to present to the Practitioner and Student of Medicine original, concise, and complete monographs on all the principal subjects of Medicine and Surgery, both general and special.

It is hoped that the series will enable the Practitioner to keep abreast with the rapid advances at present being made in medical knowledge, and that it will supplement for the Student the comparatively scanty information on special subjects contained in the general text-books.

The Series will form a complete Encyclopædia of Medical and Surgical Science in separate volumes.

The Manuals will be written by leading Hospital Physicians and Surgeons, whose work on each special subject may be considered to be authoritative.

The Manuals will be printed in clear type upon good paper. They will be of a size convenient for the pocket, substantially bound in blue cloth limp, with blue edges. Each volume will contain about 544 pages, and will be freely Illustrated by Original Chromo-Lithographs and Woodcuts, when required, and will be published at about 8s. 6d. each.

*LIST OF MANUALS.*

1.—***Insanity, including Hysteria.*** By George H. Savage, M.D., Medical Superintendent and Resident Physician to Bethlem Royal Hospital, and Lecturer on Mental Diseases at Guy's Hospital. **8s. 6d.**

2.—***Intestinal Obstruction.*** By Frederick Treves, F.R.C.S., Surgeon to and Lecturer on Anatomy at the London Hospital. **8s. 6d.**

3.—***Syphilis.*** By Jonathan Hutchinson, F.R.S., F.R.C.S., Consulting Surgeon to the London Hospital and to the Royal London Ophthalmic Hospital.

4.—***Diseases of the Breast.*** By Thomas Bryant, F.R.C.S., Surgeon to and Lecturer on Surgery at Guy's Hospital.

5.—***Surgical Diseases of the Kidney.*** By Henry Morris, M.B., F.R.C.S., Surgeon to and Lecturer on Surgery at Middlesex Hospital.

6.—***The Pulse.*** By W. H. Broadbent, M.D., F.R.C.P., Physician to and Lecturer on Medicine at St. Mary's Hospital.

7.—***Diseases of the Tongue.*** By H. T. Butlin, F.R.C.S., Assistant Surgeon to St. Bartholomew's Hospital.

8.—***Surgical Diseases of Children.*** By Edmund Owen, M.B., F.R.C.S., Surgeon to the Children's Hospital, Great Ormond Street, and Surgeon to and Lecturer on Anatomy at St. Mary's Hospital.

9.—***Diseases of the Urethra.*** By Clement Lucas, B.S., M.B., F.R.C.S., Senior Assistant Surgeon to Guy's Hospital.

10.—***Diseases of Joints.*** By Howard Marsh, F.R.C.S., Senior Assistant Surgeon to and Lecturer on Anatomy at St. Bartholomew's Hospital, and Surgeon to the Children's Hospital, Great Ormond Street.

11.—***Fractures and Dislocations.*** By T. Pickering Pick, F.R.C.S., Surgeon to and Lecturer on Surgery at St. George's Hospital.

12.—***Diseases of the Rectum and Anus.*** By Charles B. Ball, M.Ch. (Dublin), F.R.C.S.E., Surgeon and Clinical Teacher at Sir P. Dun's Hospital.

*Other Volumes will follow in due course.*

Cassell & Company, Limited, Ludgate Hill, London.

### THE YEAR-BOOK OF TREATMENT.

*A Critical Review for Practitioners of Medicine.*

Price 5s.

---

THE object of this book is to present to the Practitioner not only a complete account of all the more important advances made in the Treatment of Disease, but to furnish also a Review of the same by a competent authority.

Each department of practice will be fully and concisely treated, and into the consideration of each subject will enter such allusions to recent pathological and clinical work as bear directly upon Treatment.

The medical literature of all countries will be placed under contribution, and the work will deal with all matters relating to Treatment that have been published during the year ending September 30th.

*A full reference will be given to every article noticed.*

LIST OF THE SUBJECTS AND CONTRIBUTORS :—

1. Diseases of the Heart and Circulation. By J. MITCHELL BRUCE, M.D., F.R.C.P., Physician to Charing Cross Hospital.
2. Diseases of the Lungs and Organs of Respiration. By R. DOUGLAS POWELL, M.D., F.R.C.P., Physician to Middlesex Hospital.
3. Diseases of the Nervous System. By A. DE WATTEVILLE, M.D., B.SC., Physician to the Electrical Department, St. Mary's Hospital.
4. Diseases of the Stomach, Intestines, Liver, &c. By T. LAUDER BRUNTON, M.D., F.R.C.P., F.R.S., Assistant Physician to St. Bartholomew's Hospital.
5. Diseases of the Kidney, Diabetes, &c. By C. H. RALFE, M.D., F.R.C.P., Assistant Physician to the London Hospital.
6. Rheumatism and Gout. By DYCE DUCKWORTH, M.D., F.R.C.P., Assistant Physician to St. Bartholomew's Hospital.

7. **Anæmia, Scurvy, &c.** By SIDNEY COUPLAND, M.D., F.R.C.P. Physician to Middlesex Hospital.

8. **Medical Diseases of Children.** By A. E. SANSOM, M.D., F.R.C.P., Physician to the North Eastern Hospital for Children and to the London Hospital.

9. **Continued Fevers.** By F. A. MAHOMED, M.D., F.R.C.P., Physician to the London Fever Hospital and Assistant Physician to Guy's Hospital.

10. **General Surgery.** By THOMAS BRYANT, F.R.C.S., Surgeon to Guy's Hospital, and FREDERICK TREVES, F.R.C.S., Surgeon to the London Hospital.

11. **Orthopædic Surgery.** By J. WARRINGTON HAWARD, F.R.C.S., Surgeon to St. George's Hospital.

12. **The Surgery of the Female Pelvic Organs.** By J. KNOWSLEY THORNTON, M.B. and C.M., Surgeon to the Samaritan Hospital.

13. **Diseases of the Genito-urinary Organs.** By REGINALD HARRISON, F.R.C.S., Surgeon to the Royal Infirmary, Liverpool.

14. **Surgical Diseases of Children.** By EDMUND OWEN, F.R.C.S., Surgeon to the Children's Hospital, Great Ormond Street, and to St. Mary's Hospital.

15. **Venereal Diseases.** By ALFRED COOPER, F.R.C.S., Surgeon to the Lock Hospital.

16. **Diseases of Women.** By JOHN WILLIAMS, M.D., F.R.C.P., Obstetric Physician to University College Hospital.

17. **Midwifery.** By FRANCIS H. CHAMPNEYS, M.B., F.R.C.P., Assistant Obstetric Physician to St. George's Hospital.

18. **Diseases of the Skin.** By MALCOLM MORRIS, F.R.C.S.Ed., Surgeon to the Skin Department, St. Mary's Hospital.

19. **Diseases of the Eye.** By HENRY POWER, F.R.C.S., Ophthalmic Surgeon to St. Bartholomew's Hospital.

20. **Diseases of the Ear.** By GEORGE P. FIELD, M.R.C.S., Aural Surgeon to St. Mary's Hospital.

21. **Diseases of the Throat.** By FELIX SEMON, M.D., M.R.C.P., Physician to the Throat Department, St. Thomas's Hospital.

22. **Summary of the Therapeutics of the Year.** By WALTER G. SMITH, M.D., F.K.Q.C.P.I., King's Professor of Materia Medica, School of Physic, Trinity College, Dublin.

*Cassell & Company, Limited, London ; and all Booksellers.*

### Authoritative Work on Health by Eminent Physicians and Surgeons.

### The Book of Health.

A Systematic Treatise for the Professional and General Reader upon the Science and the Preservation of Health . *21s.*

CHAP. CONTENTS.

1. Introductory. By W. S. SAVORY, F.R.S.
2. Food and its Use in Health. By SIR RISDON BENNETT, M.D., F.R.S.
3. The Influence of Stimulants and Narcotics on Health. By T. LAUDER BRUNTON, M.D., F.R.S.
4. Education and the Nervous System. By J. CRICHTON-BROWNE, LL.D., M.D.
5. The Influence of Exercise on Health. By JAMES CANTLIE, F.R.C.S.
6. The Influence of Dress on Health. By FREDERICK TREVES, F.R.C.S.
7. The Influence of our Surroundings on Health. By J. E. POLLOCK, M.D.
8. The Influence of Travelling on Health. By J. RUSSELL REYNOLDS, M.D., F.R.S
9. Health at Home. By SHIRLEY MURPHY, M.R.C.S.
10. Health in Infancy and Childhood. By W. B. CHEADLE, M.D.
11. Health at School. By CLEMENT DUKES, M.D.
12. The Eye and Sight. By HENRY POWER, F.R.C.S.
13. The Ear and Hearing. By G. P. FIELD, M.R.C.S.
14. The Throat and Voice. By J. S. BRISTOWE, M.D., F.R.S.
15. The Teeth. By CHARLES S. TOMES, F.R.S.
16. The Skin and Hair. By MALCOLM MORRIS.
17. Health in India. By SIR JOSEPH FAYRER, K.C.S.I., F.R.S., and J. EWART, M.D.
18. Climate and Health Resorts. By HERMANN WEBER, M.D.

Edited by MALCOLM MORRIS.

"A volume which deserves high praise throughout, and which will find its uses in every household."—*The Times.*

"The work is what it aims to be—authoritative, and must become a standard work of reference not only with those who are responsible for the health of schools, workshops, and other establishments where there is a large concourse of individuals, but to every member of the community who is anxious to secure the highest possible degree of healthy living for himself and for his family."—*Lancet.*

*Cassell & Company, Limited, London; and all Booksellers.*

**Important Work on Sanitation.**

### *Our Homes, and How to Make them Healthy.*

With numerous Practical Illustrations. Edited by SHIRLEY FORSTER MURPHY, *Medical Officer of Health to the Parish of St. Pancras; Hon. Secretary to the Epidemiological Society, and to the Society of Medical Officers of Health.* 960 pages. Royal 8vo, cloth . . . . . ***15s.***

CONTENTS.

**Health in the Home.** By W. B. RICHARDSON, M.D., LL.D., F.R.S.

**Architecture.** By P. GORDON SMITH, F.R.I.B.A., and KEITH DOWNES YOUNG, A.R.I.B.A.

**Internal Decoration.** By ROBERT W. EDIS, F.S.A., and MALCOLM MORRIS, F.R.C.S.Ed.

**Lighting.** By R. BRUDENELL CARTER, F.R.C.S.

**Warming and Ventilation.** By DOUGLAS GALTON, C.B., D.C.L., F.R.S.

**House Drainage.** By WILLIAM EASSIE, C.E., F.L.S., F.G.S.

**Defective Sanitary Appliances and Arrangements.** By PROF. W. H. CORFIELD, M.A., M.D.

**Water.** By PROF. F. S. B. FRANÇOIS DE CHAUMONT, M.D., F.R.S.; ROGERS FIELD, B.A., M.I.C.E.; and J. WALLACE PEGGS, C.E.

**Disposal of Refuse by Dry Methods.** By THE EDITOR.

**The Nursery.** By WILLIAM SQUIRE, M.D., F.R.C.P.

**House Cleaning.** By PHILLIS BROWNE.

**Sickness in the House.** By THE EDITOR.

**Legal Responsibilities.** By THOS. ECCLESTON GIBB.

&c. &c.

"A large amount of useful information concerning all the rights, duties, and privileges of a householder, as well as about the best means of rendering the home picturesque, comfortable, and, above all, wholesome."—*Times.*

---

*Fourth and Cheap Edition.* Price 1s. 6d.; cloth, 2s.

### *A Handbook of Nursing*

For the Home and for the Hospital. By CATHERINE J. WOOD, *Lady Superintendent of the Hospital for Sick Children, Great Ormond Street.*

---

CASSELL & COMPANY'S COMPLETE CATALOGUE, *containing particulars of several Hundred Volumes, including Bibles and Religious Works, Illustrated and Fine-Art Volumes, Children's Books, Dictionaries, Educational Works, History, Natural History, Household and Domestic Treatises, Science, Travels, &c., together with a Synopsis of their numerous Illustrated Serial Publications, sent post free on application.*

*Cassell & Company, Limited, London; and all Booksellers.*

### Bristol Medico-Chirurgical Journal.

### MARCH, 1885.

### CONTENTS.

Medical men wishing to join the Society are requested to communicate wih the Honorary Secretary, Dr. R. SHINGLETON SMITH, 9 Richmond Hill, Clifton. The Annual Subscription is 10/6.

*Extracts from Jounal By-laws.*

4.—The *Journal* shall be delivered free to Subscribers and also to Members of the Society whose subscriptions are not in arrear.

6.—The Annual Subscription to the *Journal* for those who pay before the 1st of July shall be 5/-, post free, or 6/- if paid after that date.

Communications intended for insertion in the *Journal,* Books for Review, &c., should be sent to the Editor, J. GREIG SMITH, M.A., F.R.S.E., 16 Victoria Square, Clifton.

Exchange Journals and communications referring to the delivery of the *Journal* should be sent to the Publication Secretary, L. M. GRIFFITHS, 9 Gordon Road, Clifton, to whom subscriptions are to be paid *in advance.*

Authors requiring Reprints of their Papers should communicate with the Publisher.

*Cases for binding, price 7d. each, may be had of the Publisher,* J. W. ARROWSMITH, *Quay Street, Bristol.*

